# Agreement of the Newly Developed Moyers' Equations and Moyers' Tables

**DOI:** 10.1055/s-0042-1749157

**Published:** 2022-10-28

**Authors:** Sasipa Thiradilok, Praeopailin Witayabusarakhum, Somchai Manopatanakul, Christopher Ho

**Affiliations:** 1 Department of Advanced General Dentistry, Faculty of Dentistry, Mahidol University, Thailand; 2 Private Dentist, Bangkok, Thailand; 3 Children's Oral Health Service, Queensland Children's Hospital, Brisbane, Australia

**Keywords:** agreement test, Moyers' equations, Moyers' tables, mixed dentition analysis

## Abstract

**Objective** This study aimed to develop the Moyers' prediction equation to be used with tooth widths predicting app on smartphone.

**Materials and Methods** Four equations were developed separately for sex and dental arches. Internal validation with Moyers' table was finished. External validation on 37 subjects with agreement test of both Moyers' prediction *equations* and Moyers' prediction *tables* was performed.

**Statistical Analysis** A general linear model procedure was used to create four prediction equations. Internal validation was evaluated using the coefficient of determination. External validation was performed using Bland and Altman (BA) test.

**Results** Four equations were developed for OrthoAnalysis app on smartphone. The overall coefficient of determination of all equations and prediction table was 0.998 ( *p* < 0.05) indicating good agreement of the two methods. The agreement test on the 37 subjects was that the BA test revealed the BA limits of agreement between the residuals of two predictions was −0.001 mm and ranged from -0.143 to 0.140 mm with almost all plots lying inside this difference interval.

**Conclusions** In summary, four novel estimation equations were developed and showed very low difference to the well accepted original Moyers' prediction tables. Therefore, the equations used in the orthodontic app for predicting unerupted tooth width were verified and valid for clinical use.

## Introduction

Determination of the tooth width-arch length relationship is an important aspect of diagnosis in the mixed dentition. The tooth width-arch length discrepancy will determine whether the case will be treated with prevention of space loss or space regain. Further, in a more severe crowing situation, it also predicts whether treatment may involve extraction of some permanent teeth. Such decisions may be made preceding the eruption of the permanent canines and premolars but requires accurate mesio-distal (MD) tooth width prediction. Various analyses predicting the MD width of the pre-erupted canines and premolars in the mixed dentition have been developed. Among all these prediction methods, the Moyers' prediction table is widely used. 1 2

At this stage of advanced computer technology, health-care related apps on smartphones and mobile devices have become increasingly popular, 3 4 5 6 7 however, mostly without adequate health-care profession validation. 8 Orthodontic apps to predict tooth width may have good potential for use in daily practice. To develop a tooth width estimation app, precise tooth width prediction equations are required. 9 Beside Moyers' prediction tables, Moyers' estimation equation has not been calculated as far as the authors are aware. Therefore, it is the aim of this study to develop the Moyers' prediction equation and to test the precision of these newly developed Moyers' equations.

## Materials and Methods

All methods initiated upon completion of approval by the Institutional Review Board of the Faculty of Dentistry/Faculty of Pharmacy, Mahidol University, Thailand (COA.No.MU-DT/PY-IRB 2014/023.2306). To develop these equations, the combined widths of the canine and premolars (Moyers' tables) were first regressed on combined mandibular incisor widths (Moyers' tables) using a general linear model procedure on a Microsoft Excel program. Moyers showed the heterogeneity of the tooth size data due to gender differences and in both the maxillary and mandibular arches. Four tables were developed by Moyers based on gender and dental arch. Hence, four prediction equations were formulated accordingly, using the canine and premolar widths from the original Moyers' tables ( Tables 1 and 2 ). The correlation statistic was used to evaluate the agreement of the result from the equations and the original values from Moyers' tables. 1

**Table 1:** Predicted width of the maxillary canine and premolars from the developed Moyers' equations compared with the original Moyers' values. Male summation of tooth widths is in the upper part of the table and female in the lower part. Only two values with asterisks show that the predicted widths from the developed equations were different from Moyers' table by 0.1 mm

Males														
Sum of lower incisors(mm)		19.5	20.0	20.5	21.0	21.5	22.0	22.5	23.0	23.5	24.0	24.5	25.0	25.5
Predicted sum of canine and premolar widths (mm)	This study	20.3	20.5	20.8	21.0	21.3	21.5	21.8	22.0	22.3	22.5	22.8	23.0	23.3
	Moyers' textbook (4 ^th^ ed)	20.3	20.5	20.8	21.0	21.3	21.5	21.8	22.0	22.3	22.5	22.8	23.0	23.3
Females														
Sum of lower incisors(mm)		19.5	20.0	20.5	21.0	21.5	22.0	22.5	23.0	23.5	24.0	24.5	25.0	25.5
Predicted sum of canine and premolar widths (mm)	This study	20.3*	20.5	20.6	20.8	20.9	21.1*	21.2	21.3	21.5	21.6	21.8	21.9	22.1
	Moyers' textbook (4 ^th^ ed)	20.4	20.5	20.6	20.8	20.9	21.0	21.2	21.3	21.5	21.6	21.8	21.9	22.1

**Table 2:** Predicted outcomes of the mandibular canine and premolars from the developed Moyers' equations compared with the original Moyers' values. Male summation of tooth widths is in the upper part of the table and female in the lower part. From 26 values only four new predicted values differ from the original Moyers' values and are denoted with asterisks. All these four predictions show the difference of only 0.1 mm

Males														
Sum of lower incisors(mm)		19.5	20.0	20.5	21.0	21.5	22.0	22.5	23.0	23.5	24.0	24.5	25.0	25.5
Predicted sum of canine and premolar widths (mm)	This study	20.4	20.6	20.8	21.0	21.2	21.4	21.7*	21.9	22.1	22.3	22.5	22.7*	23.0
	Moyers' textbook (4 ^th^ ed)	20.4	20.6	20.8	21.0	21.2	21.4	21.6	21.9	22.1	22.3	22.5	22.8	23.0
Females														
Sum of lower incisors(mm)		19.5	20.0	20.5	21.0	21.5	22.0	22.5	23.0	23.5	24.0	24.5	25.0	25.5
Predicted sum of canine and premolar widths (mm)	This study	19.6	19.8	20.1	20.3	20.6	20.8	21.1	21.4*	21.6	21.9	22.1	22.4	22.6*
	Moyers' textbook (4 ^th^ ed)	19.6	19.8	20.1	20.3	20.6	20.8	21.1	21.3	21.6	21.9	22.1	22.4	22.7

To perform an external validation, 37 pairs of dental models were selected from a group of Thai subjects with normal occlusion in the permanent dentition. MD tooth widths were measured for each model. The average age of patients was 18.5 years, ranging from 11 to 30 years. The inclusion criteria were described in detail in the published article. 10 To examine the intraexaminer measurement error, two examiners repeated the measurements on 10 pairs of models. The first and second measurements of each examiner were compared using paired *t* -test and revealed no statistically significant difference ( *p* > 0.05). To examine the interexaminer measurement error, the measurements of the two examiners were also compared using similar statistical analysis. The result was also not statistically significant ( *p* > 0.05). Validation of the agreement of the results between the Moyers' table and the newly constructed formulas was undertaken using the test-retest method (Bland and Altman test [BA]). The residuals from both methods, which were the difference between the prediction result and the real tooth width, were put into the BA test. 11 12 13 14

## Results

With the use of a Microsoft Excel Program, four equations were developed based on Moyers' tables.

Maxillary teeth for boy: y = 0.5x + 10.526923076923200

Maxillary teeth for girl: y = 0.284615384615394x + 14.7961538461535

Mandibular teeth for boy: y = 0.43516483516485x + 11.87032967032970

Mandibular teeth for girl: y = 0.515384615384601x + 9.50384615384639

The results of these new equations were also calculated and compared with the original Moyers' table ( Tables 1 and 2 ). Male maxillary teeth showed the same results as Moyers' table. The male mandibular teeth and female maxillary and mandibular teeth predictions, however, showed the marginal errors of 0.1 mm. From the 13 values provided by Moyers' tables for each group, two values from these newly developed equations showed this marginal error in the maxillary arch and four values in the mandibular arch.

**Fig. 1:**
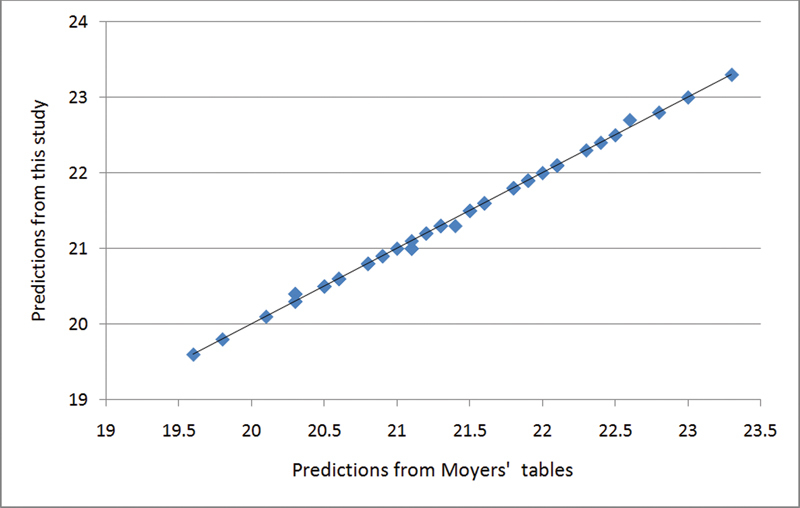
Correlation plot of the predictions from Moyers' table and the developed equations. Legend: Correlation plot of the predictions from Moyers' table (abscissa) and predictions from the newly developed equations (ordinate). Both abscissa and ordinate coordinate's distribution showed strong positive correlation.

**Fig. 2:**
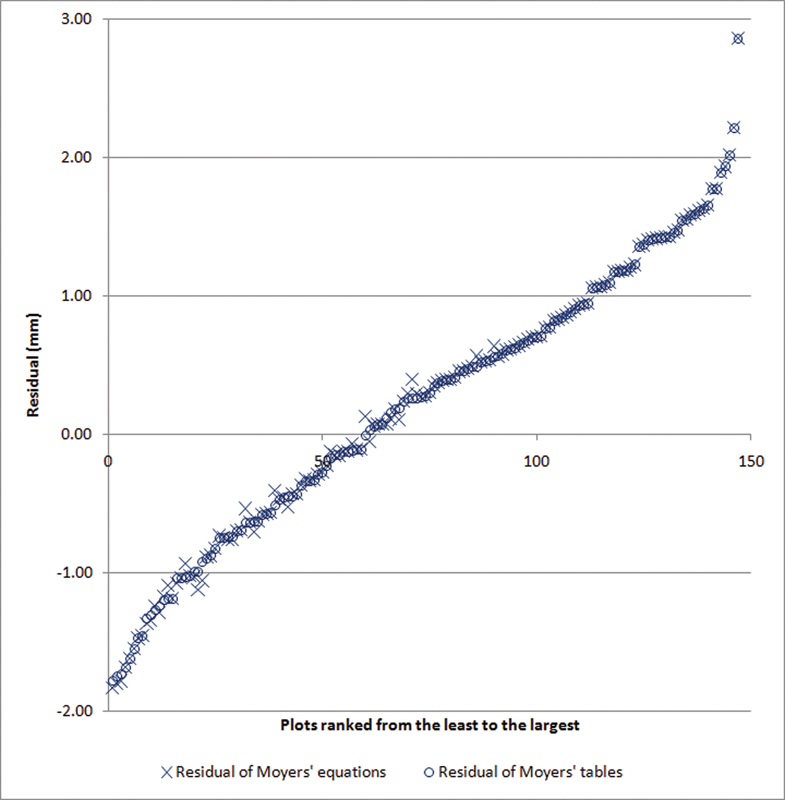
Residual plots between the two methods. All 148 residuals of the two methods were sorted from the least to the largest and plotted along the x-axis, using a cross symbol for Moyers' equation and a circle symbol for the Moyers' table. The y-axis showed the unit of difference from actual tooth width in mm. The plots of both predictions distributed very close to each other in almost every individual pair reflecting an agreement of these two prediction methods.

**Fig. 3:**
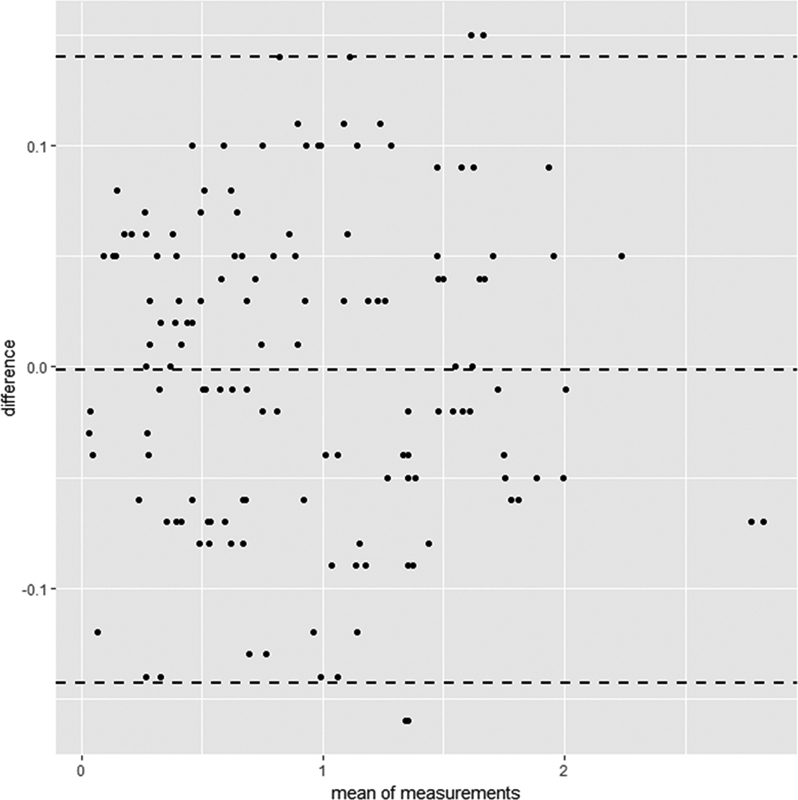
Bland and Altman (BA) plot of the difference of residuals of both prediction methods. Legend: BA plot of the difference of residuals that is the difference between the prediction result and the real tooth width of both prediction methods. The averages of the residuals of two methods were plotted against the x-axis. The differences of the residuals of the two methods were plotted against the y-axis. The dotted line represented the 95% limit of agreement (LoA) showing the limit of the acceptability of the difference. The BA LoA between the residuals of these two prediction methods was only −0.001 mm and ranged from −0.143 to 0.140 mm with almost all plots lying inside the difference interval.

The overall correlation coefficient between all equations and Moyers' prediction tables was 0.998 ( *p* < 0.05). The scatter plot, derived from the correlation of these two predictions methods, showed congruent results as the agreement of these two methods was high ( Fig. 1 ). The difference of the prediction and the real MD width of canine and premolars of the four quadrants of 37 subjects were demonstrated in Fig. 2 . All plots of both prediction methods distributed close to each other. The agreement test on all subjects was also completed ( Fig. 3 ). The BA test revealed the BA limits of agreement (LoA) between the residuals of these two prediction methods was only −0.001 mm and ranged from −0.143 to 0.140 mm with almost all plots lying inside the difference interval.

## Discussion

Many methods have been used to simplify the predicted width of pre-erupted canine and premolars. Pancherz and Schäffer suggested the application of a unitary prediction value, 22.0 mm, for every German patient from the central part of Hessen. 15 Even though Pancherz and Schäffer postulated that 22.0 mm, which is the mean value plus one standard deviation, performed better than Moyers' 75th percentile estimation, this simplification was not applicable in subjects with smaller and larger tooth sizes. This study, however, developed simple equations with high precision that may be applied to any computer system based on Moyers' tables.

Moyers' equations

Many tooth width estimation methods were developed for orthodontic space analysis. Moyers' prediction has been widely used. To provide a simple calculation, it is important to develop formulae for use with new technology to calculate this estimation. With the use of the slope and intercept calculation from a Microsoft Excel program, these equations were calculated. As there are many types of equations that could be produced for Moyers' equation, the simple linear regression was chosen. The function called Trend/Regression type was selected and applied to the best-fit straight line to display simple linear datasets with the condition that these sets of values increase or decrease in a steady rate. The calculated least squares fit for this trend line was also calculated using the linear equation,



where *m* is the slope and *b* is the intercept. In addition, *b* is calculated as:



The function in the Excel program made it simple to calculate the unerupted tooth widths prediction equations.

However, there may be some doubt on the agreement of these equations to Moyers' tables. There was a suggestion to increase the precision of this equation by maximizing the number of decimal places. 16 Hence, the maximum number of decimal places was used. To check the comparability of using the equations to Moyers' prediction tables, the outcome of these equations was verified by statistically comparing them to the original Moyers' tables. The results showed agreement between the two predictions especially for maxillary male teeth. The mandibular male, the maxillary and mandibular female teeth, however, showed marginal error of 0.1 mm for two values from each equation.

In addition, external validation of these equations was tested with tooth widths from 37 Thai subjects. The difference between the prediction and real tooth widths of these two estimation methods was very similar ( Fig. 2 ). The very small difference of −0.001 mm with the BALoA of −0.143 to 0.140 mm was considered clinically insignificant ( Fig. 3 ). One must also consider that the original Moyers' tables were developed from the northern European population. Moyers recommended different percentile for adjustment of predicted value due to practitioners' experience. Although the specific table developed from the particular ethnic group is the most precise, 17 many studies modified the percentile of Moyers's tables, showing adequate reproducibility to their population. 18 19 20 21 In this study, there was no attempt to adjust the percentile even though Moyers' tables were applied to a Thai ethnic group. This is due to the fact that even without the percentile adjustment, the agreement of both predictions was still very high. However, care should be taken when these equations are applied to the different racial groups. Future research on a larger Thai sample size and different ethnic groups is planned to further test these equations.

Since Moyers reported four tables predicting the unerupted tooth width separately, this study proposed four novel Moyers' equations accordingly. These four equations provided the width of maxillary and mandibular combined canine-premolars crown width in each gender. Further, the development of an orthodontic app for smartphones facilitates orthodontists in predicting unerupted tooth size. This app, developed by Witayabusarakhum et al, utilized these prediction equations to provide reliable results for tooth width prediction. 9

There is shortage in educational and clinical diagnosis medical and dental apps. Further, orthodontist requires reminder and strategized behavioral app. Although there were increasing in number of the health-care apps, there is a need for a call to action for health-care professions involvement in app development and validation. 8

## Conclusions

To facilitate the calculation of predicted canine and premolars tooth widths based on the tooth widths of the mandibular incisors, four novel estimation equations were developed using a Microsoft Excel program. Results from the equations were compared with Moyers' tables and statistically verified. The results of these two prediction methods showed very low difference and had high agreement. The equations were verified and assured for use in an orthodontic app. This study also promotes the orthodontists contribution in app creation and validation.
